# Cover Picture: Metastable β-Bi_2_O_3_ Nanoparticles with Potential for Photocatalytic Water Purification Using Visible Light Irradiation (ChemistryOpen 4/2013)

**DOI:** 10.1002/open.201390015

**Published:** 2013-08-26

**Keywords:** bismuth oxide nanoparticles, bismuth oxido clusters, organic pollutants, photocatalysis, visible light irradiation

## Abstract

**Cover Picture:**

Maik Schlesinger, Marcus Weber, Steffen Schulze, Michael Hietschold, and Michael Mehring*

**The cover picture shows** the degradation of the model dye rhodamine B (RhB) in water using β-Bi_2_O_3_ nanoparticles as photocatalyst, which was prepared from preorganized bismuth oxido clusters. In the background, a homemade photoreactor for in situ studies is shown, including the decolorizing process of the aqueous RhB solution as observed during visible light irradiation. The catalytic cycle shows crucial steps in RhB degradation at the surface of β-Bi_2_O_3_ nanoparticles (balls), which can be recycled. After adsorption of RhB at the surface of β-Bi_2_O_3_ nanoparticles, the color of the nanoparticles changes from orange to violet. The degradation process (represented by Pac-Man) is induced by irradiation with visible light, resulting in a complete decolorization of the solution as well as the surface of the β-Bi_2_O_3_ nanoparticles, indicating a fast de-ethylation process. The latter process is faster than desorption of partially degraded RhB species from the β-Bi_2_O_3_ surface, but is demonstrated to occur upon irradiation of RhB-loaded β-Bi_2_O_3_ in the solid state. For more details, see the Full Paper by Michael Mehring et al., on p. 146 ff.

